# Superparamagnetic core/shell GoldMag nanoparticles: size-, concentration- and time-dependent cellular nanotoxicity on human umbilical vein endothelial cells and the suitable conditions for magnetic resonance imaging

**DOI:** 10.1186/s12951-015-0080-x

**Published:** 2015-03-25

**Authors:** Mingfu Gong, Hua Yang, Song Zhang, Yan Yang, Dong Zhang, Yueyong Qi, Liguang Zou

**Affiliations:** Department of Radiology, Xinqiao Hospital, Third Military Medical University, Chongqing, China

**Keywords:** Superparamagnetic, Core/shell GoldMag nanoparticles, Human umbilical vein endothelial cells, Cellular nanotoxicity, Reactive oxygen species, Labeling efficiency, Magnetic resonance imaging

## Abstract

**Background:**

GoldMag nanoparticles (GMNPs) possess the properties of colloid gold and superparamagnetic iron oxide nanoparticles, which make them useful for delivery, separation and molecular imaging. However, because of the nanometer effect, GMNPs are highly toxic. Thus, the biosafety of GMNPs should be fully studied prior to their use in biomedicine. The main purpose of this study was to evaluate the nanotoxicity of GMNPs on human umbilical vein endothelial cells (HUVECs) and determine a suitable size, concentration and time for magnetic resonance imaging (MRI).

**Results:**

Transmission electron microscopy showed that GMNPs had a typical shell/core structure, and the shell was confirmed to be gold using energy dispersive spectrometer analysis. The average sizes of the 30 and 50 nm GMNPs were 30.65 ± 3.15 and 49.23 ± 5.01 nm, respectively, and the shell thickness were 6.8 ± 0.65 and 8.5 ± 1.36 nm, respectively. Dynamic light scattering showed that the hydrodynamic diameter of the 30 and 50 nm GMNPs were 33.2 ± 2.68 and 53.12 ± 4.56 nm, respectively. The *r*_*2*_ relaxivity of the 50 nm GMNPs was 98.65 mM^−1^ s^−1^, whereas it was 80.18 mM^−1^ s^−1^ for the 30 nm GMNPs. The proliferation, cytoskeleton, migration, tube formation, apoptosis and ROS generation of labeled HUVECs depended on the size and concentration of GMNPs and the time of exposure. Because of the higher labeling rate, the 50 nm GMNPs exhibited a significant increase in nanotoxicity compared with the 30 nm GMNPs at the same concentration and time. At no more than 25 μg/mL and 12 hours, the 50 nm GMNPs exhibited no significant nanotoxicity in HUVECs, whereas no toxicity was observed at 50 μg/mL and 24 hours for the 30 nm GMNPs.

**Conclusions:**

These results demonstrated that the nanotoxicity of GMNPs in HUVECs depended on size, concentration and time. Exposure to larger GMNPs with a higher concentration for a longer period of time resulted in a higher labeling rate and ROS level for HUVECs. Coupled with *r*_*2*_ relaxivity, it was suggested that the 50 nm GMNPs are more suitable for HUVEC labeling and MRI, and the suitable concentration and time were 25 μg/mL and 12 hours.

## Background

Because of their small size and high surface area to volume ratio, nanoparticles exhibit unusual physicochemical properties, which have led to their use in sports equipment, the photovoltaic industry, industrial catalysis and electronics. Additionally, nanoparticles are smaller than a cell, protein or gene, which has led to their increased use in biomedicine for the purpose of drug delivery, molecular imaging and targeting therapy. The increasingly greater application of nanomaterials in our daily life has aroused public concerns regarding adverse effects on humans. Because of the higher surface area to volume ratio, higher surface reactivity and susceptibility to degradation and ion leaching, nanoparticles are generally considered to have higher levels of toxicity compared with bulk material in some studies [1-3]. Furthermore, through interactions with specific biomolecules, nanoparticles can induce noxious molecules, such as reactive oxygen species (ROS), which cause apoptosis in cells and interfere with organ function [4,5]. Thus, the biosafety of nanoparticles should be fully studied prior to their use in biomedicine.

Magnetic resonance imaging (MRI) is one of the best noninvasive methods currently used in clinical medicine because of its superb soft-tissue contrast resolution, lack of radiation exposure, and multi-parameter and multi-sequence imaging [6]. MRI is less sensitive compared with positron emission tomography and fluorescence imaging; therefore, MRI cannot be used for small lesion monitoring or molecule tracing [7]. However, contrast agents (CAs) markedly enhance the sensitivity of MRI. Superparamagnetic iron oxide nanoparticles (SPIO) exhibit extremely high magnetic moments in the presence of an external magnetic field, markedly shortening the transverse relaxation time (T_2_) and T_2_^*^, which is of great interest for researchers; this technology has the potential to provide improvements in the field of molecular imaging, gene and drug delivery and cell trafficking [8-10].

GoldMag nanoparticles (GMNPs) are a type of composite nanoparticle that have a typical shell/core structure, with SPIO as the core and a layer of gold coating the surface [11]. Gold is a noble metal, which displays a strong optical absorbance and localized surface Plasmon resonance, ensuring that gold is a superb candidate for surface enhanced Raman scattering as well as chemical and biological sensors. Additionally, proteins could be conjugated to gold nanoparticles relatively easily through thiol chemistry or physisorption. Furthermore, the excellent biocompatibility of gold, which is derived from its lack of toxicity and chemical inertness coupled with its rapid heating by near infrared irradiation, ensures that it is an excellent candidate for biomedical applications [12,13]. GMNPs possess the properties of colloid gold and SPIO; as a result, GMNPs have been used in the delivery, separation and purification of biological samples and are an excellent candidate for multimodal molecular imaging [14,15].

There is a close relationship between angiogenesis and tumor growth, invasion, metastasis, as well as the prognosis of cancer. The diameter of a tumor does not exceed 2–3 mm in the absence of a blood vessel [16]. Hematogenous dissemination of cancer cells is the primary route for distant metastasis of malignant tumors [17]. The direct or indirect assessment of tumor blood vessels is crucial to identify tumor occurrence, development and prognosis. Endothelial cells are the leading target in the study of tumor angiogenesis. Combining the real-time and highly sensitive visualization of MR molecular imaging, MR targeted imaging of endothelial cells labeled with GMNPs is an ideal platform to assess angiogenesis.

In previous studies, GMNPs have been applied to MR imaging and other biomedical applications [11,14]. Most studies have explored the feasibility of MR imaging based on GMNPs, and a limited number evaluated nanotoxicity on biological systems. In this study we used GMNPs of two sizes to label human umbilical venous endothelial cells (HUVECs), and we evaluated the labeling efficiency and nanotoxicity of GMNPs. In addition, we explored the preliminary nanotoxic mechanism of GMNPs. We identified a suitable size, concentration and duration of GMNP labeling of HUVECs for targeted MRI of cells.

## Results

### GMNP characterization

**Figure 1:**
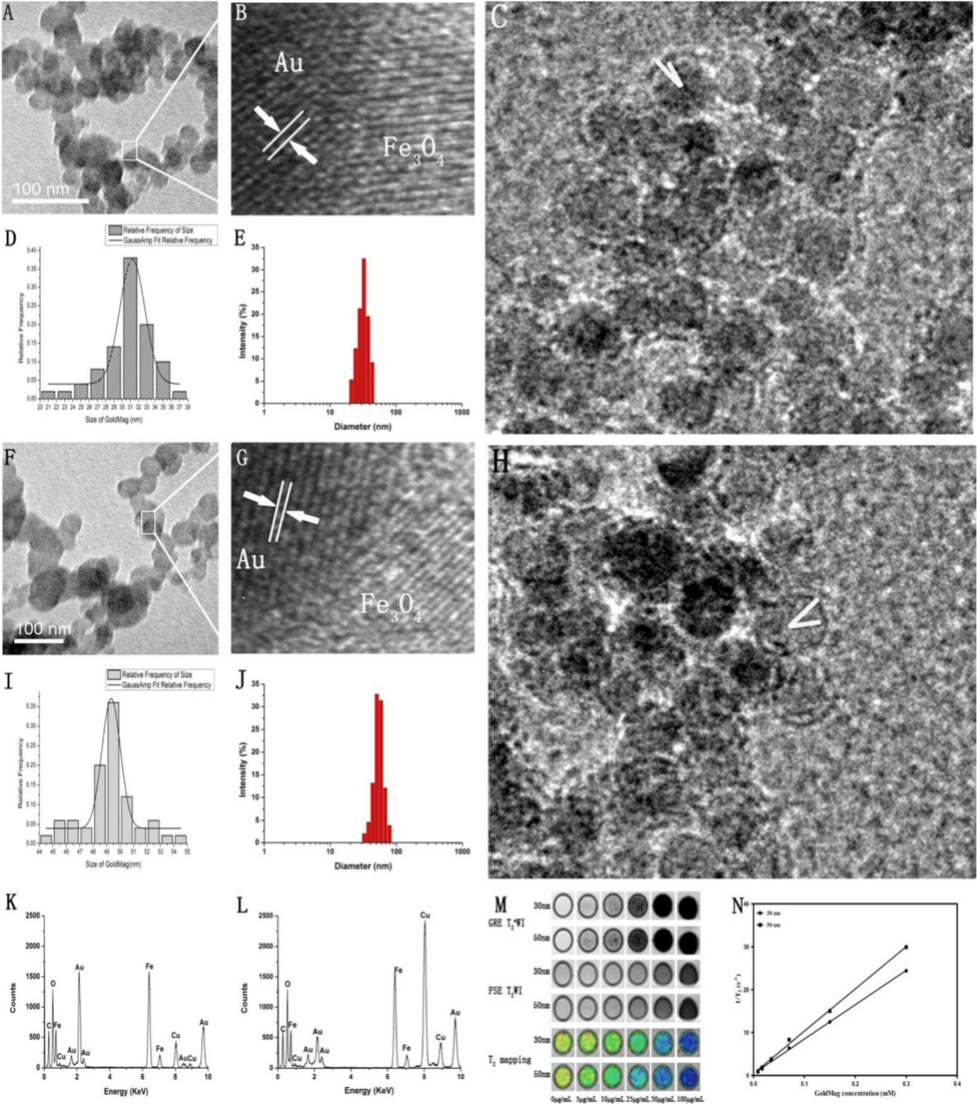
**GoldMag nanoparticles characterization.** TEM, HRTEM, size distribution histograms, hydrodynamic diameter and EDS of 30 nm **(** **A,** **B,** **C,** **D,** **E** and **K** **)** and 50 nm **(** **F,** **G,** **H,** **I,** **J** and **L** **)** GMNPs. MR images of 30 nm and 50 nm GMNPs with different concentrations; the r_2_ relaxivity of each are shown in **(M)** and **(N)**, respectively.

The photomicrographs obtained by transmission electron microscopy (TEM) showed that the average particle sizes of the 30 and 50 nm GMNPs were 30.65 ± 3.15 (Figure 1A and D) and 49.23 ± 5.01 nm (Figure 1 F and I), respectively, as counted from 100 randomly selected nanoparticle; the nanoparticles had a fairly spherical shape and a relatively narrow particle size distribution. The magnified TEM images showed that there was a high electron density coating on the surface of GMNPs (Figure 1C and H), indicating the presence of an Au shell on the surface of Fe_3_O_4_ nanoparticles. The core size and the gold shell thickness of the 30 nm GMNPs were 23.3 ± 1.95 nm and 6.8 ± 0.65 nm, respectively (Figure 1C), and these values were 38.2 ± 2.3 nm and 8.5 ± 1.36 nm, respectively, for the 50 nm GMNPs (Figure 1H). High resolution transmission electron microscopy (HRTEM) images of 30 nm GMNPs and 50 nm GMNPs are shown in Figure 1B and Figure 1G respectively. The spacing between the lattice fringes was measured to be approximately 0.24 nm which correspond to the plane (111) of Au, also indicating the deposition of Au on the Fe_3_O_4_ nanoparticles. The hydrodynamic diameter of GMNPs dispersed in water was determined by dynamic light scattering (DLS). Figures 1E and J show that the average hydrodynamic diameters of the 30 nm and 50 nm GMNPs were 33.2 ± 2.68 nm and 53.12 ± 4.56 nm, respectively, which is in good agreement with the TEM results. The energy dispersive spectrometer (EDS) spectrum of 30 nm GMNPs (Figure 1K) and 50 nm GMNPs (Figure 1L) revealed the presence of Au, Fe and O in the GMNPs, whereas the Cu and C signal were derived from the copper grid that was used to prepare the TEM sample. The GMNPs showed a concentration-dependent signal drop in the GRE T_2_^*^WI and FSE T_2_WI. The GMNPs that were 50 nm in size induced greater hypo-intensities at the identical concentrations compared with that of the 30 nm GMNPs. The linear fitting showed that the *r*_*2*_ relaxivity of the 50 nm GMNPs was 98.65 mM^−1^ s^−1^, which is 1.23 times higher than that of the 30 nm GMNPs (80.18 mM^−1^ s^−1^) (Figure 1 M-N).

### Uptake of the GMNPs

**Figure 2:**
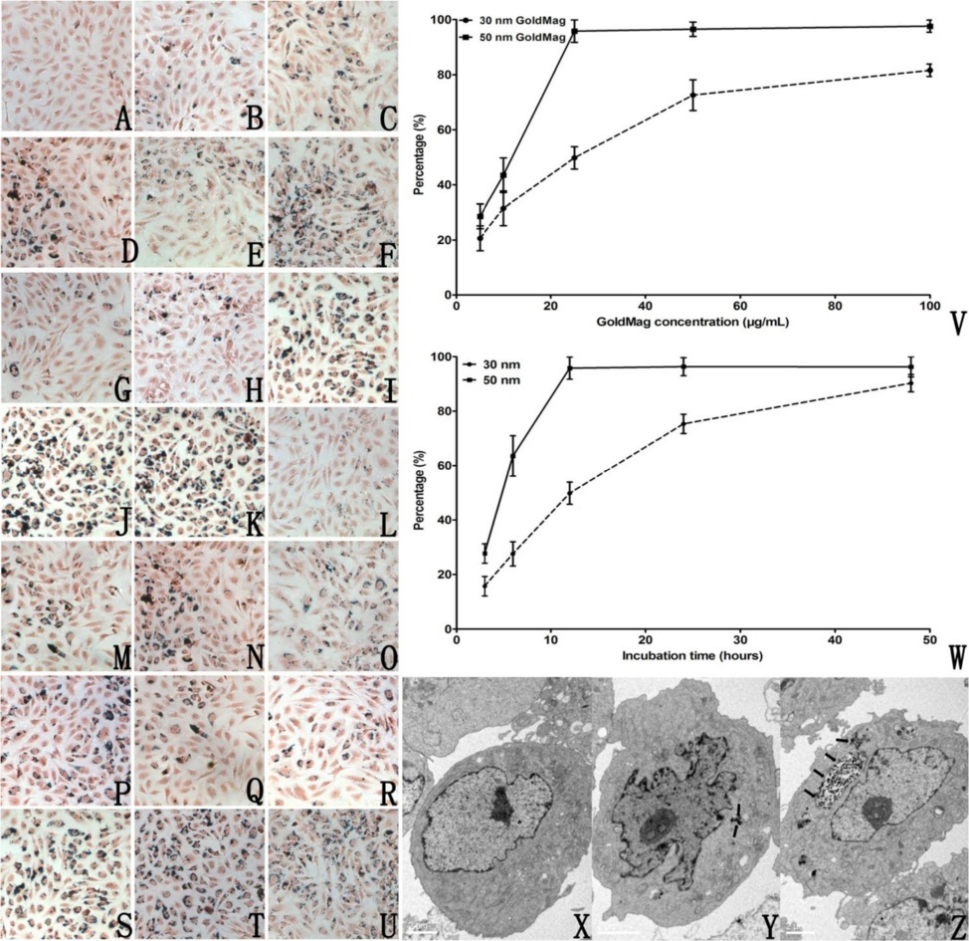
**GMNP uptake.** Prussian blue staining of HUVECs **(** **A**-**U,** x200**)**. Cells were incubated with 5, 10, 25, 50 and 100 μg/mL 30 nm GMNPs for 12 hours **(** **B**-**F** **)**, 5, 10, 25, 50 and 100 μg/mL 50 nm GMNPs for 12 hours **(** **G**-**K** **)**, 25 μg/mL 30 nm GMNPs for 3, 6, 12, 24 and 48 hours **(** **L** **-** **P** **)**, and 25 μg/mL 50 nm GMNPs for 3, 6, 12, 24 and 48 hours **(** **Q** **-** **U** **)**. Control cells are shown in **(A)**. The labeling rates of 30 and 50 nm GMNPs at different concentrations and for durations are shown in **(** **V** **-** **W** **)**. TEM of control HUVECs **(X)**, HUVECs labeled with 25 μg/mL 30 nm GMNPs **(** **Y,** black arrow**)** and 50 nm GMNPs **(** **Z,** black arrow**)** for 12 hours.

In this study, the uptake of the GMNPs was determined using TEM and Prussian blue staining. Figure 2X-Z shows that the untreated and treated HUVECs are oval, spindle or irregular polygons with a complete cell structure. Some vacuoles were within the HUVECs exposed to the GMNPs. The vacuoles contained round, electron-dense particles, which were indicative of the presence of the GMNPs sequestered within the labeled HUVECs. These vesicles were distributed in the perinuclear region and did not penetrate the nucleus or the mitochondria. Compared with the 30 nm GMNPs, there were more vacuoles and the scale of the vacuoles was larger in the cells labeled with the 50 nm GMNPs, which indicates that there were more 50 nm GMNPs engulfed than 30 nm GMNPs.

Because SPIO can produce ferric ferricyanide, a dark blue pigment known as Prussian blue, through its reaction with potassium ferrocyanide within the acidic solution, the GMNPs could be observed with an optical microscope after Prussian blue staining. Figure 2A-U clearly showed that there were blue granules in the cytoplasm of the labeled cells and most of them were around the nucleus, which is perfectly consistent with the TEM results. The uptake of the GMNPs depended on the size, time and concentration of the GMNPs. With an increasing concentration and co-incubation time, the number of cells containing blue particles and blue granules in each cell increased. The labeling rate of the HUVECs labeled with 50 nm GMNPs is higher than that of the cells labeled with 30 nm GMNPs at the identical concentration and exposure time. Specifically, the labeling rate of the 50 nm GMNPs was 95.8% after co-incubation with 25 μg/mL of GMNPs for 12 hours, which was 50.2% for the 30 nm GMNPs at the identical exposure.

### Cell proliferation, apoptosis, cytoskeleton, migration and tube formation

**Figure 3:**
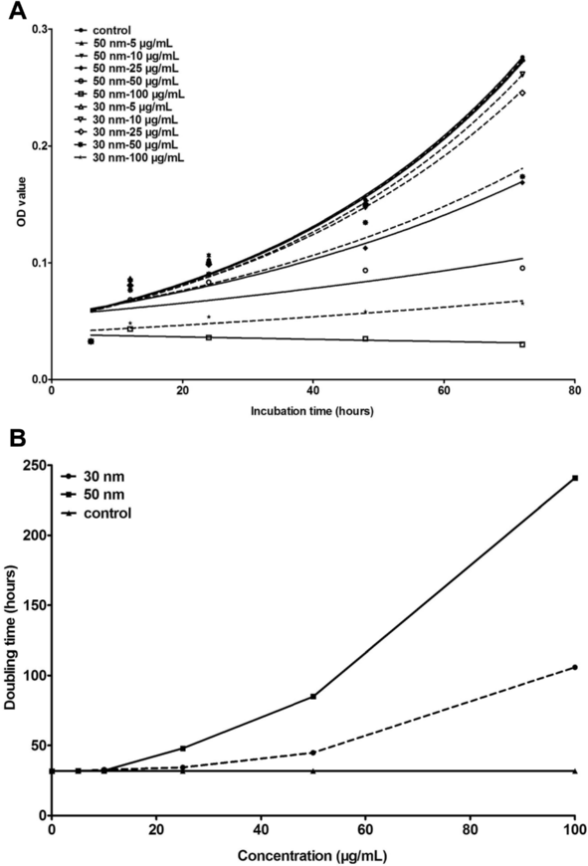
**Growth curve of HUVECs exposed to different concentrations of 30 and 50 nm GMNPs (A) and the doubling time of these HUVECs (B).** HUVEC proliferation was affected by the GMNPs in a size-, concentration- and time-dependent manner.

According to the growth curve based on the optical density (OD) value and exposure time, the doubling time of the untreated HUVECs is 31.81 hours, which is very close to the doubling time of the cells exposed to low concentrations of 30 and 50 nm GMNPs (<25 μg/mL). With increasing concentration and exposure time, the doubling time of the labeled HUVECs rapidly increased. Under the identical concentration and exposure time, the doubling time of the HUVECs labeled with 30 nm GMNPs was much shorter than that of the cells labeled with 50 nm GMNPs (Figure 3). HUVEC proliferation was affected by the GMNPs in a size-, concentration- and time-dependent manner. For more than a specific concentration and exposure time of the GMNPs (25 μg/mL and 48 hours for 50 nm and 50 μg/mL and 72 hours for 30 nm), the OD value was below that at 0 hours, indicating that the GMNPs were toxic and caused noticeable cell necrosis.

**Figure 4:**
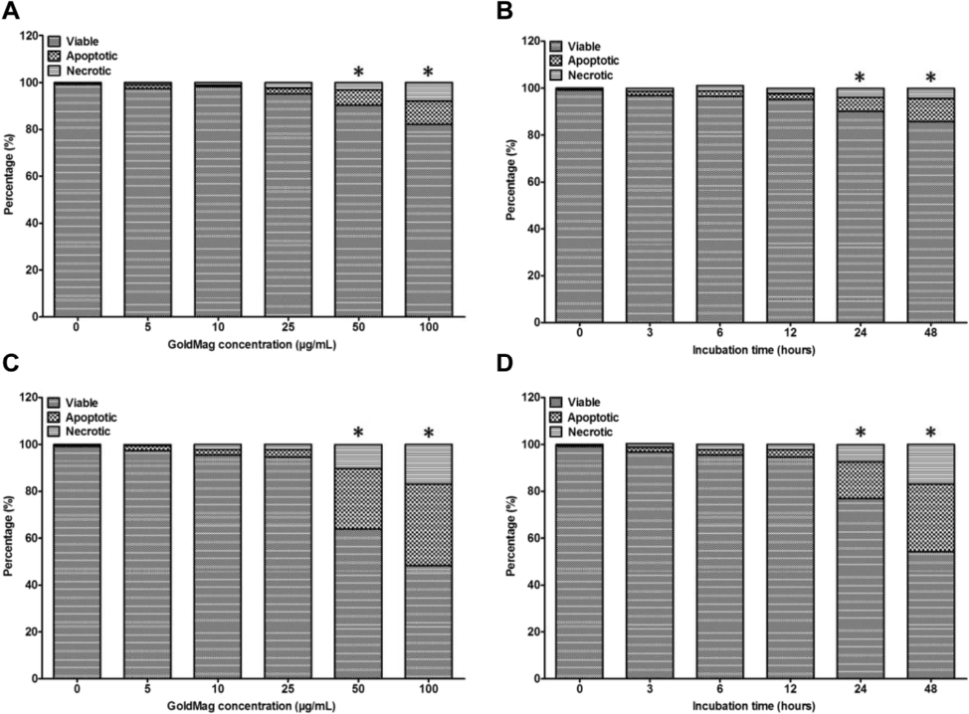
**HUVEC apoptosis. (A)** and **(B)** HUVECs labeled with 30 nm GMNPs; **(C)** and **(D)** for HUVECs labeled with 50 nm GMNPs. ^*^ *P* < 0.05, compared with the control group.

The Annexin V-FITC apoptosis analysis showed that for the 50 nm GMNPs, a significant decrease of approximately ~24% in the viability of cells incubated with 25 μg/mL nanoparticles for 24 hours was measured compared with the controls; for the 30 nm GMNPs, a decrease of only approximately 10% in the viability of the HUVECs was measured compared with the controls at the identical dose and time. Considering all the cells labeled with GMNPs of different sizes, concentrations, and duration, the proportion of the apoptotic cells was a function of the time and concentration for the 30 nm and 50 nm GMNPs, whereas the 50 nm GMNPs caused a larger proportion of the cells to undergo apoptosis than the 30 nm GMNPs at the identical dose and time. There was a significant increase in the number of apoptotic cells detected only after exposure to at least 25 μg/mL 50 nm GMNPs for more than 12 hours and at least 50 μg/mL 30 nm GMNPs for a minimum of 24 hours (Figure 4).

**Figure 5:**
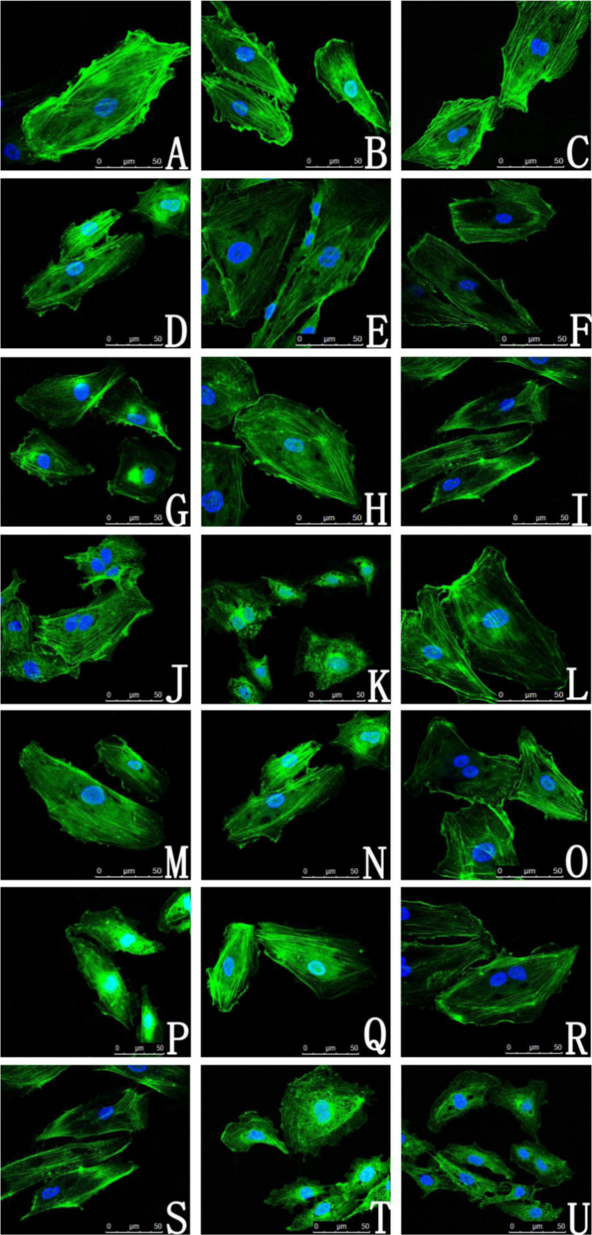
**CLSM of HUVEC cytoskeletons. (A)** Control; **(** **B** **-** **F** **)** HUVECs labeled with 5, 10, 25, 50 and 100 μg/mL 30 nm GMNPs for 12 hours; **(** **G** **-** **K** **)** HUVECs labeled with 5, 10, 25, 50 and 100 μg/mL 50 nm GMNPs for 12 hours; **(L-P)** HUVECs labeled with 25 μg/mL 30 nm GMNPs for 3, 6, 12, 24 and 48 hours; **(** **Q** **-** **U** **)** HUVECs labeled with 25 μg/mL 50 nm GMNPs for 3, 6, 12, 24 and 48 hours. The cytoskeletons of the cells exposed to the 30 and 50 nm GMNPs at either 50 or 100 μg/mL for more than 24 hours exhibited a fractured, corrugated, and sparse appearance. The round, electron-dense vacuoles lacking fluorescence and sequestered perinuclearly within these HUVECs were verified to be GMNPs.

The cytoskeleton is a cellular skeleton that provides cells with structure and shape; it plays important roles in many cellular behaviors, such as intracellular transport and cellular division. Therefore, the integrity of the cytoskeleton structure and function is very important for cells. Here, our group observed the cytoskeleton and morphology of the labeled cells by staining them with fluorescent phalloidin under confocal laser scanning microscope (CLSM). We determined that the untreated HUVECs stretched well and adhered to the wall with a clear and smooth cytoskeleton distributed uniformly within the cells. The cells, which were incubated with the 50 nm GMNPs at 5, 10 or 25 μg/mL for less than 24 hours, exhibited a similar appearance compared with the control cells, which the exception of some round, electron-dense vacuoles; these vacuoles lacked fluorescence sequestered within these HUVECs perinuclearly and were verified to be GMNPs under the white light view of CLSM. A clear loss of the cytoskeleton network could be observed when the cells were exposed to the 50 nm GMNPs at either 50 or 100 μg/mL for more than 24 hours. Under a high magnification view of these HUVECs, the cytoskeleton exhibited a fractured, corrugated, and sparse appearance. Furthermore, some cells became necrotic and dissolved, and their cytoskeleton became disorganized. It was clear that the effects on the HUVEC cytoskeleton architecture that were induced by the 50 nm GMNPs were size-, concentration- and exposure time-dependent. The overall trend of reducing the cytoskeleton network of the HUVECs labeled with the 30 nm GMNPs, according to the concentration and incubation time, was similar; however, the smaller size resulted in a reduction in the inhibition effects and less loss of the cytoskeleton architecture under the identical concentration and exposure time compared with the 50 nm GMNPs. The highest, nontoxic concentration for the cytoskeleton after treatment with the 30 nm GMNPs was higher compared with the 50 nm GMNPs after 12 hours of exposure; these concentrations were 50 μg/mL and 25 μg/mL, respectively (Figure 5).

**Figure 6:**
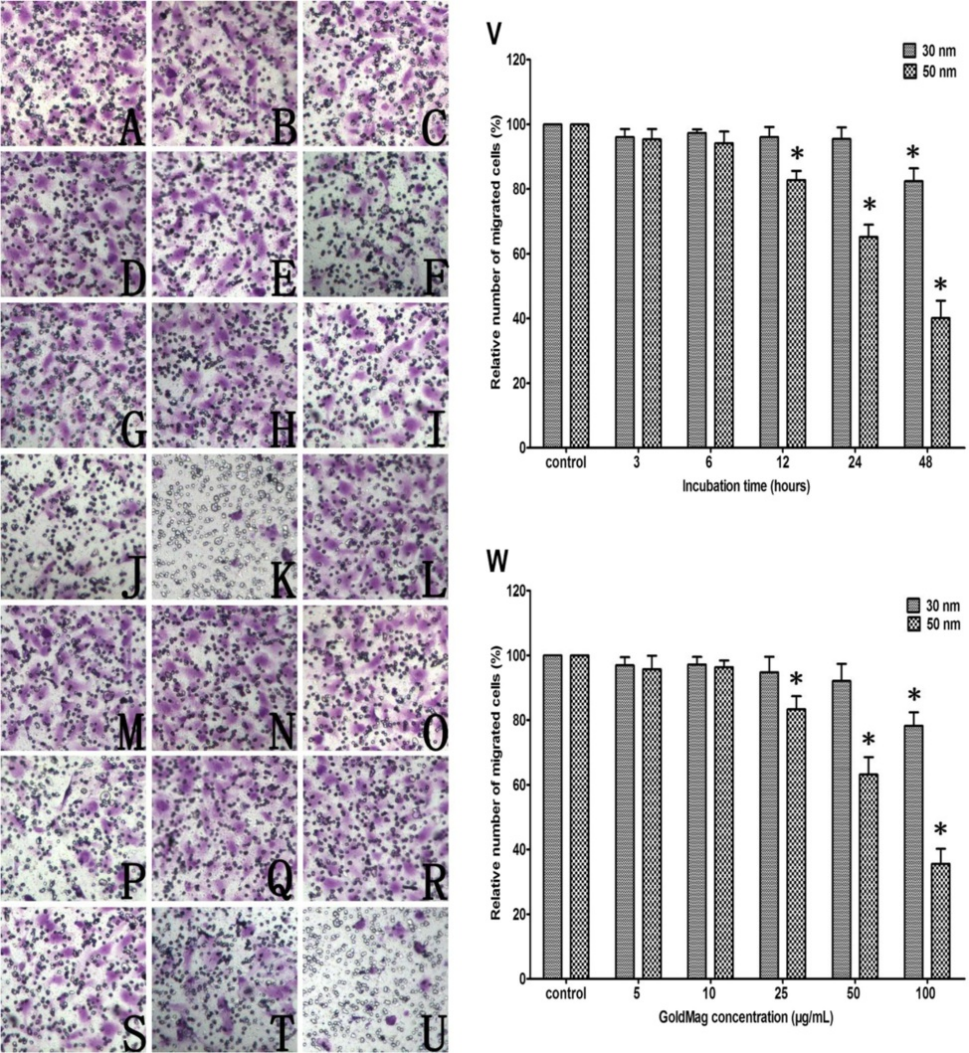
**Crystal violet staining of migrated HUVECs (A-U, x200). (A)** Control; **(** **B** **-** **F** **)** HUVECs labeled with 5, 10, 25, 50 and 100 μg/mL 30 nm GMNPs for 12 hours; **(** **G** **-** **K** **)** HUVECs labeled with 5, 10, 25, 50 and 100 μg/mL 50 nm GMNPs for 12 hours; **(** **L** **-** **P** **)** HUVECs labeled with 25 μg/mL 30 nm GMNPs for 3, 6, 12, 24 and 48 hours; **(** **Q** **-** **U** **)** HUVECs labeled with 25 μg/mL 50 nm GMNPs for 3, 6, 12, 24 and 48 hours. **(** **V** **-** **W** **)** Relative number of migrated HUVECs labeled with different concentrations of 30 and 50 nm GMNPs for various times. ^*^ *P* < 0.05, compared with the control group.

Cell migration is a complex cellular behavior that is precisely regulated by the cytoskeleton and other regulatory proteins. The results from the transwell assay showed that the migration of labeled HUVECs was affected by the GMNPs in a size-, concentration- and time-dependent manner. With increased concentration and incubation time, the 30 and 50 nm GMNPs began to exhibit HUVEC migration-inhibiting activity at 25 μg/mL for 48 hours and 25 μg/mL for 12 hours, respectively. Compared with the control group, the average relative number of the migrated HUVECs treated with 25 μg/mL of the 30 nm GMNPs for 48 hours and the 50 nm GMNPs for 12 hours were 81.4 ± 4.1% and 83.6 ± 2.9%, respectively. Compared with the 30 nm GMNPs, the 50 nm GMNPs exhibited much higher inhibition of migration at the identical concentration (Figure 6).

**Figure 7:**
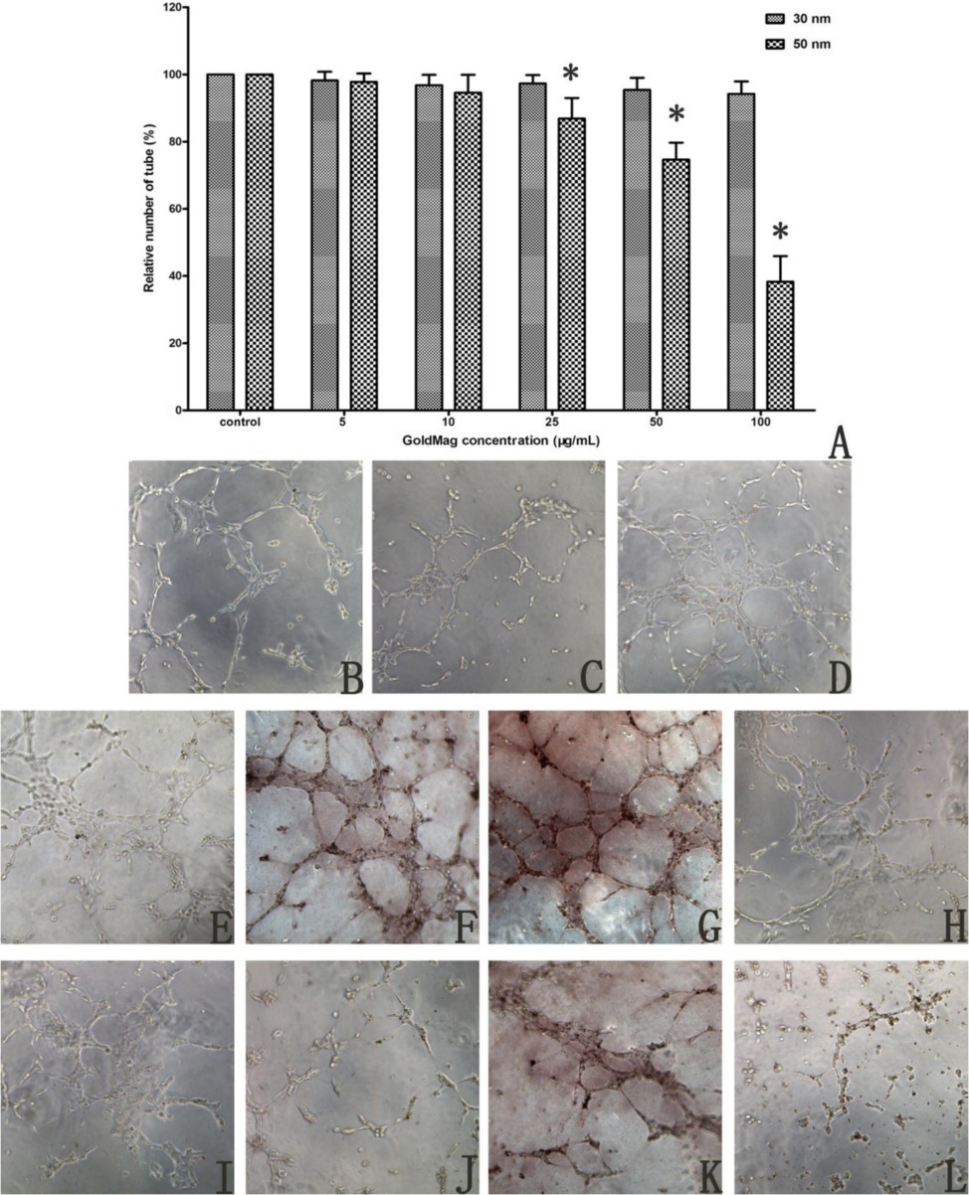
**Photographs of tube-like structures (B-L, x100). (B)** Control; **(** **C** **-** **G** **)** HUVECs labeled with 5, 10, 25, 50 and 100 μg/mL 30 nm GMNPs; **(** **H** **-** **L** **)** HUVECs labeled with 5, 10, 25, 50 and 100 μg/mL 50 nm GMNPs. **(A)** Relative number of tube-like structures formed from HUVECs labeled with different concentrations of 30 and 50 nm GMNPs for 12 hours, ^*^ *P* < 0.05, compared with the control group.

Angiogenesis, the formation of new capillaries from existing vasculature, is crucial for physiological and pathological events, including wound healing, inflammation and the growth and progression of tumors. The formation of tube-like structures from endothelial cells under appropriate conditions is a critical step in angiogenesis. In this study, a tube formation assay was used to assess the biotoxicity of the GMNPs using a three-dimensional Matrigel assay. For the control cells, elongated tube-like structures were formed 4 hours after cell adhesion, which were also observed for the cells that were labeled with low concentration GMNPs. Fewer tube structures were formed from the cells that were incubated with 100 μg/mL of 30 nm GMNPs and 25 μg/mL of 50 nm GMNPs. Additionally, higher concentrations were associated with more GMNP-inhibited tube formation. Under identical labeling conditions, the 50 nm GMNPs exhibited higher inhibition ability compared with the 30 nm GMNPs (Figure 7).

### ROS generation

**Figure 8:**
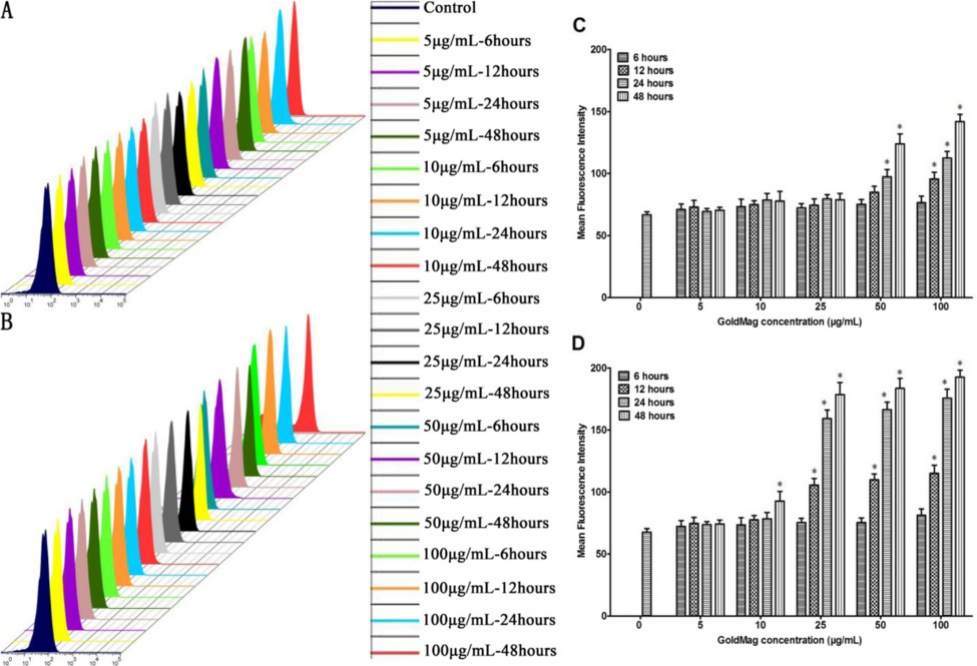
**ROS generation in HUVECs. (A)** and **(C)** for HUVECs labeled with 30 nm GMNPs; **(B)** and **(D)** for HUVECs labeled with 50 nm GMNPs. ^*^ *P* < 0.05, compared with the control group. ROS induction depended on size, concentration and time. A significant elevation in the ROS was detected with increasing size, concentration and time.

As shown in Figure 8, the induction of ROS depended on the size, concentration and time. For the cells labeled with the 50 nm GMNPs, a significant elevation in the ROS was detected in the cells that were exposed to 25 μg/mL of GMNPs for 24 hours, which was 2.5 times higher than that of the control cells. Similar results were obtained for the HUVECs that were labeled with the 30 nm GMNPs, reaching a significant increase in the intracellular ROS at 50 μg/mL for 24 hours. At short incubation times and low labeling concentrations, minimal intracellular ROS elevations were observed compared with those of the control cells.

### In vitro MRI of HUVECs

**Figure 9:**
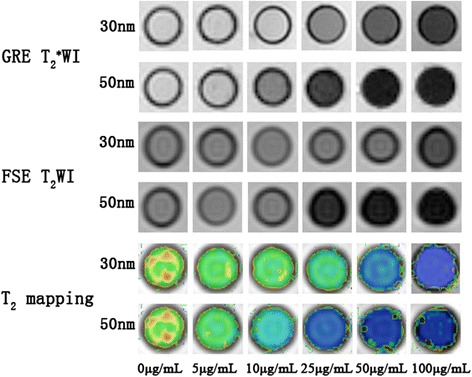
**MR images of HUVECs labeled with different concentrations of 30 and 50 nm GMNPs.** With increasing concentrations, the signal intensity of the HUVECs labeled with the 30 and 50 nm GMNPs decreased rapidly. Consistent with the trend of the signal intensity, the T_2_ relaxation time of the labeled cells decreased with increasing GMNP concentration.

With increasing concentration, the signal intensity for the T_2_ weighted and T_2_^*^ weighted MRI of the HUVECs labeled with the 30 nm and 50 nm GMNPs decreased rapidly. Consistent with the trend of the signal intensity in the T_2_-weighted MRI, the T_2_ relaxation time of the labeled cells decreased with an increased GMNP concentration. Compared with the cells that were labeled with the 30 nm GMNPs, the T_2_ relaxation time of the HUVECs labeled with 50 nm GMNPs was much lower. The cells incubated with 100 μg/mL of the 30 nm and 50 nm GMNPs showed the shortest T_2_ relaxation times, which were 19.68 ms and 10.76 ms, respectively (Figure 9).

## Discussion

The uptake of nanoparticles is a prerequisite and crucial step for successful cell labeling and MRI [18]. Many studies have investigated the factors that influence nanoparticle uptake, which is a complex process that may be influenced by many factors, including the nanoparticle size, shape, surface coating, and surface charge. Because particles of different sizes possess distinct optical, electronic, and magnetic properties, the uptake of nanoparticles is considered to most likely be size-dependent, and many reports on the uptake and toxicity of nanoparticles have focused on the particle size [19-23]. In previous research, the uptake of many types of nanoparticles, including quantum dots, liposomes, gold, silver, silica and iron oxide nanoparticles, was reported to be size-dependent [24-26]. In the study of Clift et al. [22] study, the uptake of 20 nm nanoparticles by macrophages was easier, faster and more extensive compared with that of 200 nm nanoparticles, which was hypothesized to be because of different uptake mechanisms for differentially sized nanoparticles. Huang et al. [19] evaluated the uptake of gold nanoparticles that ranged from 2 to 15 nm for monolayer breast cancer cells and obtained a similar result, in which 2 nm nanoparticles exhibited levels of higher cellular uptake than 6 and 15 nm nanoparticles because of their ultra-small structure. Additional studies [4,20,23] have drawn a similar conclusion that a higher level of nanoparticle uptake was concomitant with a decrease in nanoparticle size. Concurrent with this finding, a recent hypothesis is that the uptake of nanoparticles is size-dependent, with the optimal size for cell uptake equal to 50 nm [21,27,28]. Lu et al. [21] investigated the uptake of various sizes of silica nanoparticles, which ranged from 30 to 280 nm, and discovered that the cellular uptake was highly size dependent with 50 > 30 > 110 > 280 > 170 nm. Ma et al. [28] reported that the cellular uptake of gold nanoparticles was heavily dependent on the particle size, and 50 nm Au nanoparticles were most readily internalized by cells, followed by 25 nm and 10 nm Au nanoparticles. In this study, the internalization of GMNPs was verified by Prussian blue staining and TEM (Figure 2). The number of GMNPs ingested by HUVECs was concentration- and time-dependent; there was a gradual increase in the number of blue-stained cells as the concentration and incubation time increased. Compared with the 30 nm GMNPs, the internalization of the 50 nm GMNPs was substantially faster and more efficient. These findings are consistent with other studies [21,27,28]. Previously, the uptake of gold nanoparticles was determined to occur via the endocytosis pathway, which is mediated by the serum protein that nonspecifically adsorbed onto the gold nanoparticle surface. The number of binding sites on the nanoparticles is dependent on the surface area, which increases with the particle size. Combined with the steric hindrance effect, the protein density on the particle surface increases linearly with size. Larger nanoparticles have more proteins on the particle surface, allowing for their more efficient internalization. Coupled with an uptake dependent on the membrane wrapping time, which is based on the diffusion rate of the receptors on the membrane, extremely large nanoparticles with substantially higher surface protein density are incapable of compensating for the depletion of receptors within the binding area and exhibit slight internalization. According to kinetics, the increasing elastic energy associated with the bending of the membrane results in a decreased driving force for the membrane wrapping of smaller nanoparticles. Smaller particles must be clustered to create sufficient driving energy for uptake, and the uptake level of smaller particles is substantially smaller compared with that of optimally sized particles [27,28]. Furthermore, based on thiol chemistry, we hypothesize that the internalization of GMNPs is mediated by the adsorption of membrane proteins with thiol onto the surface of GMNPs, which results in production similar to receptor-mediated endocytosis.

An overwhelming majority of reports state that iron oxide nanoparticles are biosafe because the Fe ions are biocompatible and highly tolerated [29,30]. Additionally, a study by Huang reports that the ionic SPIO could diminish the intracellular H_2_O_2_ through intrinsic peroxidase-like activity and accelerate cell cycle progression to promote cell growth [29]. Some reports have confirmed that gold is biosafe because of its chemical inertness [4,31,32]. However, other researchers have demonstrated that these nanoparticles are not inherently benign and have potential toxicity at the cellular, subcellular and protein levels in a size-, time- and dose-dependent manner [3,5,33,34]. In this study, a multi-parametric study was conducted to systematically assess the cytotoxic effects of the GMNPs on the HUVECs. Considering all of the results, GMNPs have cytotoxic effects that depend on the concentration and incubation time; the bioactivity of the HUVECs that were incubated with a relatively low concentration of GMNPs for a short duration was not significantly changed, and gradual cytotoxicity was observed when the HUVECs were labeled with 50 μg/mL of 30 nm GMNPs and 25 μg/mL of 50 nm GMNPs for 12 hours. Extremely high concentrations of GMNPs could induce HUVEC apoptosis and necrosis (Figures 3, 4, 5, 6 and 7). It is difficult to explain the exact mechanism by which GMNPs exhibit noticeable cytotoxic effects. However, according to a previous study regarding nanoparticle toxicity, several factors may be responsible for these effects, including ROS production, genotoxicity, morphological modifications and toxic ion leaching; ROS induction has been posited as one of the main explanations for these effects [5,33-35]. Particles within the nanometer range might lead to structural defects and could damage the electronic configuration, which may create electron donor or acceptor sites on the nanoparticle surface. Molecular dioxygen (O_2_) surrounding these nanoparticles would react with the reactive sites and lead to the formation of a superoxide radical (O^2−^). Additionally, many free hydroxyl radicals (OH^−^) are generated through Fenton chemistry and Haber-Weiss reactions under the catalysis of transition metals (e.g., Fe and manganese). Both OH^−^ and O^2−^, collectively known as ROS, are generally perceived as toxins that induce various deleterious effects, including cell membrane damage, DNA and cytoskeleton injury, autophagy and apoptosis [35-37]. Here, the results of the DCFH-DA assay demonstrated that the induction of ROS was clearly concentration- and time-dependent for HUVECs labeled with 30 and 50 nm GMNPs, which is in agreement with the cytotoxicity results of the GMNPs on HUVECs and can be understood based on the aforementioned theory for ROS. With increasing concentrations and times, the number of GMNPs engulfed by the cells gradually increased, resulting in the accumulation of ROS within the cells and deleterious effects on the DNA, proteins, cell membrane and cytoskeleton (Figure 8). Nevertheless, GMNPs are a composite nanoparticle that have a layer of gold coating on the surface of the SPIOs; gold is not as relevant as the transition metals with respect to nanotoxicity [32,38] because it is chemically inert. Two factors might be responsible for this contradictory phenomenon. The synthesis of GMNPs is a two-step process involving the formation of iron oxide nanoparticles and a layer of gold deposition on the surface. The synthesis is relatively complex, and there might be some uncontrollable factors that result in incomplete coating of the core with gold (Figures 1C and H). Prussian blue staining of the labeled HUVECs has also confirmed this assumption; ferric ferricyanide could only be produced by ferric iron through its reaction with potassium ferrocyanide within an acidic solution. Furthermore, gold has cytotoxicity through other mechanisms, including cell morphology and cytoskeleton defects; interactions with mitochondria; disturbances in the intracellular signaling pathways via interference with stimulating factors; and DNA damage and genotoxicity [39-41]. Additionally, some recent studies have paradoxically suggested that gold nanoparticles cause oxidative stress in mammalian cells after internalization [34,35,42].

Compared with the 30 nm GMNPs, the 50 nm GMNPs exhibited higher cytotoxicity at the same concentration and incubation time. These findings are inconsistent with reports that smaller nanoparticles have a higher surface area to volume ratio and higher surface reactivity, which results in higher nanotoxicity [1,2]. Additionally, according to the study by Meng et al. [43], smaller nanoparticles have a higher degree of curvature and generate more potential toxicity. The difference in the level of GMNPs internalized into the HUVECs might be responsible for the seemingly contradictory results. In a previous study, Soenen et al. [34] demonstrated that the induction of ROS and toxic effects of nanoparticles occurred while the cells or subcellular structures maintained contact with the nanoparticles. The results from the Prussian blue staining showed that internalization of the 50 nm GMNPs was much more likely and efficient than internalization of the 30 nm GMNPs. The presence of more nanoparticles within the cells results in the generation of more ROS and greater deleterious effects on the DNA, proteins, cell membrane and cytoskeleton, leading to higher nanotoxicity.

The main purpose of this study was to evaluate the bioactivity effects of GMNPs with different sizes, concentrations and incubation times on HUVECs, to explore the mechanisms of GMNP nanotoxicity and to select a suitable size, concentration and incubation time for in vitro and in vivo MR imaging. Thus, the relaxivity of GMNPs with different sizes should be considered. Relaxivity is defined as the increase in the nuclear relaxation rate of water protons produced by 1 mmol/L of CAs, which indicates the signal enhancement efficiency produced by the MRI CAs. The iron oxide nanoparticles within GMNPs could shorten the longitudinal relaxation time and T_2_; however, the predominant effect is on the T_2_ and T_2_^*^ shortening, which produces the darkening of the contrast-enhanced tissue. The results in this report show that both types of GMNPs showed a concentration-dependent signal drop in the GRE T_2_^*^WI, FSE T_2_WI and T_2_ mapping and that the *r*_*2*_ relaxivity of the 50 nm GMNPs is approximately 1.5 times higher than that of the 30 nm GMNPs. These findings are consistent with the majority of previous studies on this topic [44,45]; in these previous studies, the SPIO exhibit extremely high magnetic moments because of a cooperative alignment of the electronic spins of the individual paramagnetic ions, and the *r*_*2*_ relaxivity is proportional to the particle size. Combined with the uptake of GMNPs of different sizes, it was clearly demonstrated that the MRI signal drop of labeled HUVECs increased with increased concentrations of both sizes of GMNPs, and the HUVECs labeled with the 50 nm GMNPs produced higher negative enhancement compared with the 30 nm GMNPs at the same concentration.

## Conclusion

In this work, the morphology, size, size distribution and relaxivity of the GMNPs were characterized through TEM and MRI; we focused on the nanotoxicity assessment of GMNPs on HUVECs using a detailed and multiparametric approach. Various aspects of bioactivity were analyzed, including cell proliferation, cytoskeleton integrity, migration, tube formation and apoptosis. In addition, ROS generation in the HUVECs labeled with GMNPs of different sizes, concentrations and incubation times was studied to explore the mechanisms of nanotoxicity. We found that the GMNPs had a regular aspect, fairly spherical shape and relatively narrow particle size distribution. The *r*_*2*_ relaxivity of the 50 nm GMNPs is approximately 1.5 times higher than that of the 30 nm GMNPs. The uptake and bioactivity effects, including cell proliferation, the cytoskeleton, migration, tube formation, apoptosis and the generation of ROS of the labeled HUVECs, depended on size, concentration and time. Combined with the relaxivity of the GMNPs of different sizes, the 50 nm GMNPs are more suitable for HUVEC labeling and MR imaging in vitro, and the optimal labeling concentration and incubation time are 25 μg/mL and 12 hours, respectively, which produce significant negative enhancement in MRI and do not lead to significant effects with respect to cell proliferation, the cytoskeleton, migration, tube formation, apoptosis or ROS induction. In this work, we conducted a multiparametric assessment of the cytotoxicity of the GMNPs and defined a suitable size, concentration and incubation time for cell labeling and MR imaging. This work provides a reference for future studies using the same nanoparticles for cell labeling and MRI in vitro or in vivo.

## Methods

### Cell culture

HUVEC cells were used in this study, and were routinely harvested via the digestion of human umbilical veins with type-I collagenase, as previously described [46]. The human umbilical cords were obtained from the Department of Obstetrics and Gynecology of Xinqiao Hospital. The specificity and purity of the isolated cells were evaluated using immunofluorescence staining and flow cytometry (FCM). HUVECs were cultured in medium 199 (Hyclone, UT, USA) supplemented with 20% fetal bovine serum (FBS) (Hyclone), endothelial cell growth supplement (Sciencell, USA), 0.05 mg/mL heparin (Sigma, USA), 2 mM L-glutamine (Sigma), and 100 U/mL penicillin/streptomycin (Hyclone) in a humidified incubator (Thermo scientific, USA) with 5% CO_2_ at 37°C. The third to seventh passages were used for the subsequent experiments. Proliferation and tube formation assays were performed at a density of 3 × 10^3^ and 1 × 10^4^ cells/well in 96-well plates (Corning, NY, USA), respectively. HUVECs incubated with various concentrations (0, 5, 10, 25, 50 and 100 μg/mL) of GMNPs (30 and 50 nm) were used for the proliferation assay and the tube formation assay, and the time periods used for incubation were 6, 12, 24, 48 and 72 hours and 6 hours, respectively. HUVECs were cultured in glass bottom cultures with a diameter of 30 mm (NEST, China) and 6-well plates (Corning) with 1 × 10^5^ cells per culture or well for the cytoskeleton and Prussian-blue staining, migration, and apoptosis assays, which were exposed to 25 μg/mL GMNPs (30 and 50 nm) for various time periods (3, 6, 12, 24 and 48 hours) and incubated with various concentrations (0, 5, 10, 25, 50 and 100 μg/mL) of GMNPs (30 and 50 nm) for 12 hours. For TEM and MRI, HUVECs were incubated with 25 μg/mL GMNPs (30 and 50 nm) for 12 hours. HUVECs exposed to various concentrations (0, 5, 10, 25, 50 and 100 μg/mL) of GMNPs (30 and 50 nm) for various time periods (6, 12, 24 and 48 hours) were used to assess ROS generation. All cells were cultured overnight for adherence and achieved 80% confluence prior to exposure to GMNPs. For the fluorescence experiments, the cells were maintained under lucifugal conditions. Each experiment had a control and was repeated at least three times.

### TEM, EDS, DLS and MRI

GMNPs of 30 and 50 nm were purchased from Xi’an GoldMag Nanobiotech Co., Ltd. (Xi’an, China). They were prepared by the reduction of Au^3+^ with hydroxylamine in the presence of Fe_3_O_4_ particles as seeds [11]. Briefly, the Fe_3_O_4_ particles were co-precipitated from Fe (II) and Fe (III) ions in alkaline medium and then dispersed in chloroauric acid solution. NH_2_OH solution was then added to the mixture, which was incubated with shaking for 1 hour. Finally, the prepared GMNPs were washed with plenty of water. The morphology, particle size and size distribution of the GMNPs were measured using TEM (Hitachi-7500, Japan) and HRTEM (JEM-2100 F, Japan). The diameter of GMNPs in dispersion was determined using the DLS technique (Nano zs90, Malvern, England). The chemical composition of GMNPs was quantified by EDS analysis (JEM-2100 F). Following fixation, dehydration and embedding, the labeled HUVECs were cut into sections with a thickness of 60 nm using a diamond knife before using TEM to observe their ultra-structures and confirm the location of the GMNPs within them. Serial concentrations (0, 5, 10, 25, 50 and 100 μg/mL) of the GMNPs (30 and 50 nm) and labeled HUVECs were resuspended in 1% agarose gel and scanned in a head coil using a 3.0 T clinical MR scanner (Signa HDx, GE, USA). The scanning parameters were as follows: matrix 256 × 256, FOV 16 cm × 16 cm, interlayer spacing 0.4 mm, FSE T_2_WI (TR 2000 ms and TE 43.7 ms), GRE T_2_^*^WI (TR 400 ms, TE 12.0 ms, and Flip angle 30°) and 16 echo T_2_ mapping (TR 1025 ms and TE 2.4-60.5 ms). The *r*_*2*_ relaxivity of the GMNPs was calculated through the curve fitting of the 1/T_2_ relaxation time (s^−1^) vs. the concentration (mM). The region of interest was 5 mm^2^.

### Prussian blue staining

The labeled HUVECs were fixed with 4% paraformaldehyde, incubated with Prussian blue staining solution (which contained equal volumes of 2% hydrochloric acid and 2% potassium ferrocyanide) for 30 min and stained with a neutral red solution for 10 min. The images were obtained using an inverted fluorescence microscope (DMIRB, Leica, Germany).

### In vitro cell proliferation assay

The cell proliferation assay was performed with a cell Count Kit-8 (CCK-8) (Beoyotime Biotechnology Company, China). Because the CCK-8 assay relies on the OD of orange formazan and may be affected by the GMNPs, the medium that contained the GMNPs was displaced by the mixture that contained 100 μL of fresh medium and 10 μL of 2-(2-methoxy-4-nitrophenyl)-3-(4-nitrophenyl)-5-(2,4-disulfophenyl)-2H-tetrazolium after incubation for the corresponding time. Following 1.5 hours of co-culture, the medium that contained formazan was transferred into a new 96-well plate with a permanent magnet under the plate to minimize the influence of GMNPs on the absorbance. A spectral scanning multimode reader (Varioskan Flash, Thermo) was used to determine the OD at a wavelength of 490 nm.

### In vitro cytoskeleton assay

The treated HUVECs were fixed with 4% paraformaldehyde and incubated with fluorescent phalloidin (Sigma) according to the manufacturer’s procedure. Following staining with 4′,6-diamidino-2-phenylindole (DAPI) (Roche, Switzerland), the HUVECs were mounted with an anti-fluorescence quenching agent and observed under a CLSM (SP5, Leica).

### HUVEC migration assay

The migration assay was conducted in a 6.5-mm diameter transwell chamber (Millipore, USA) with 8-μm pore filters. Treated HUVECs (10^4^) in 0.1 mL of medium with 1% FBS were added to the upper compartment, and 0.6 mL of M199 with 10% FBS was added to the lower compartment to stimulate migration. After 12 hours in the culture, the HUVECs on the lower surface of the polycarbonate membrane were fixed with 4% paraformaldehyde and stained with 2% crystal violet. The migrated cells were evaluated with an inverted fluorescence microscope.

### In vitro vasculogenesis assay

The effects of the GMNPs on the in vitro differentiation of the HUVECs were evaluated through a vasculogenesis assay using an Angiogenesis Assay Kit (BD, USA). According to the manufacturer’s instructions, all labeled HUVECs were cultured as previously described and observed under an inverted light microscope every 2 hours. Five independent fields were assessed for each well, and the mean number of tubules/100× field was determined.

### Quantitation of ROS generation

The level of intracellular ROS was quantified using a ROS assay kit (Sigma). After labeling with the GMNPs, the HUVECs were incubated with 2′,7′-dichlorofluorescin diacetate (DCFH-DA) according to the manufacturer’s procedure. The fluorescent intensity of the HUVECs was measured by FCM (Moflo XDP, Beckman Coulter, USA) with excitation and emission wavelengths of 488 and 525 nm, respectively.

### Apoptosis assay

Apoptosis of the HUVECs exposed to various GMNPs was measured using the Annexin V-FITC Apoptosis Analysis Kit (Sigma) according to the manufacturer’s instructions. All samples were analyzed using FCM by measuring the average fluorescent intensity.

### Statistics

All results are expressed as the mean ± standard deviation. The statistical comparisons were performed using Student’s *t*-test and one way ANOVA; a *P* value <0.05 indicated a significant difference.

## Abbreviations

CAs: Contrast agents
CCK-8: Cell count kit-8
CLSM: Confocal laser scanning microscope
DAPI: 4′,6-diamidino-2-phenylindole
DCFH-DA: 2′,7′-dichlorofluorescin diacetate
DLS: Dynamic light scattering
EDS: Energy dispersive spectrometer
FBS: Fetal bovine serum
FCM: Flow cytometry
GMNPs: GoldMag nanoparticles
HRTEM: High resolution transmission electron microscopy
HUVECs: Human umbilical vein endothelial cells
MRI: Magnetic resonance imaging
OD: Optical density
ROS: Reactive oxygen species
SPIO: Superparamagnetic iron oxide nanoparticles
T_2_: Transverse relaxation time
TEM: Transmission electron microscopy
